# Dose-escalation by hypofractionated simultaneous integrated boost IMRT in unresectable stage III non-small-cell lung cancer

**DOI:** 10.1186/s12885-021-09099-3

**Published:** 2022-01-22

**Authors:** Qin Zhang, Xu-Wei Cai, Wen Feng, Wen Yu, Xiao-Long Fu

**Affiliations:** Department of Radiation Oncology, Shanghai Chest Hospital, Shanghai Jiao Tong University, 241 West Huaihai Road, Shanghai, 200030 China

**Keywords:** Dose escalation, SIB-IMRT, Hypofractionated radiotherapy, Non-small cell lung cancer

## Abstract

**Background:**

To explore the maximum tolerated dose (MTD) and evaluate the safety of dose escalation using hypofractionated simultaneous integrated boost intensity-modulated radiotherapy (SIB-IMRT) concurrent with chemotherapy for unresectable stage III non-small cell lung cancer (NSCLC).

**Methods:**

Four escalating radiation dose levels were used. This study included 25 patients with previously untreated NSCLC who received six concurrent weekly chemotherapy cycles comprising cisplatin and docetaxel. Dose-limiting toxicity (DLT) was defined as any acute toxicity that interrupted radiotherapy for more than 1 week. MTD was defined as the highest dose level that didn’t induce DLT or grade 5 toxicity in two patients.

**Results:**

All 25 patients received the prescribed escalating radiation dose from the start dose up to LEVEL 4. Two patients experienced DLT at dose LEVEL 4. One patient died because of upper gastrointestinal hemorrhage within 6 months after radiotherapy, whereas another patient among the additional five patients died because of grade 5 radiation pneumonitis within 2 months after radiotherapy. Dose LEVEL 3 was defined as MTD. The 1- and 2-year local controls were 82.8 and 67.8%, respectively. The median progression-free survival was 15.4 months, whereas the median overall survival was 27.3 months.

**Conclusions:**

Dose escalation was safely achieved up to LEVEL 3 [the planning gross target volume (PTVG) 60.5 Gy/22 Fx, 2.75 Gy/Fx; the planning clinical target volume (PTVC) 49.5 Gy/22 Fx] using SIB-IMRT concurrently with chemotherapy for unresectable stage III NSCLC, and the acute toxicities were generally well tolerated. Further prospective studies on long-term outcomes and late toxicities are warranted.

**Trial registration:**

Retrospective registration, ChiCTR1900027290(08/11/2019).

**Supplementary Information:**

The online version contains supplementary material available at 10.1186/s12885-021-09099-3.

## Introduction

Approximately 85% of all newly diagnosed lung cancer is non-small cell lung cancer (NSCLC) [1, 2]. Furthermore, approximately 30% of patients have unresectable locally advanced (LA) stage III cancer [3]. The standard treatment at this stage is concurrent chemoradiotherapy (CRT), which provides a 4.5% overall survival (OS) advantage at 5 years compared with sequential CRT [4, 5]. This survival benefit is because of better locoregional control.

However, even after definitive CRT with the standard radiotherapy dose (60 Gy/30 Fx), locoregional failure occurred in up to 30–40% of unresectable stage III NSCLC patients [6]. Radiation dose escalation was proposed to solve and improve the locoregional control rate and OS and also the quality of life. Unfortunately, the higher dose 74Gy failed to get survival benefit in the Radiation Therapy Oncology Group (RTOG) 0617. Other studies provided evidence of feasibility with a suitable higher radiation dose, which was absent in RTOG 0617 [6, 7]. Therefore, it is essential to investigate the optimal dose escalation with advances in technology.

Hypofractionated radiotherapy delivers higher doses per fraction, shortens treatment time, decreases the effect of accelerated repopulation, increases biologically effective doses (BEDs), and potentially improves locoregional control [8–11]. As demonstrated with stereotactic body radiotherapy (SBRT) for stage I NSCLC, hypofractionated radiotherapy with large BEDs achieved superior locoregional control of the primary lesion without increasing adjacent normal tissue toxicity [12–18]. Studies of LA-NSCLC revealed that concurrent hypofractionated radiotherapy and chemotherapy are feasible therapeutic approaches for increasing efficacy. We previously conducted a phase II study of LA-NSCLC patients who received accelerated hypofractionated radiotherapy considering that the high dose of 68 Gy was safe and effective [9]. However, the implementation of hypofractionated radiotherapy is limited because of many side effects caused by delivering large volume doses to nearby body structures. Studies have previously verified that compared with conformal radiotherapy (3DCRT), IMRT can improve radiation dose distributions and decrease dose delivery to nearby crucial structures [19–25]. Based on intensity-modulated planning and delivery, the use of a simultaneous integrated boost (SIB) technique can simultaneously deliver a relatively higher dose to the target tumor and a lower dose to the subclinical tumor in NSCLC patients [26, 27].

Thus, as mentioned, based on a 2-Gy/Fx, 60 Gy/30 Fx as the standard dose, we designed this study to explore the maximum tolerated dose (MTD) and evaluate the safety of dose escalation using hypofractionated SIB-IMRT with concurrent chemotherapy for unresectable stage III NSCLC.

## Methods and materials

### Eligibility criteria

The eligibility criteria were as follows (Additional file 1): (1) those classified as having unresectable stage III NSCLC (according to the 7th edition American Joint Committee on Cancer); (2) those having histologically or cytologically confirmed NSCLC; (3) those not previously treated; (4) those with Eastern Cooperative Oncology Group performance status (PS) 0–1; (5) those aged between 18 and 75 years; (6) those classified with weight loss < 10%; and (7) those with OS of > 3 months. Patients who were breastfeeding, pregnant, had a history of another malignant tumor within the past 5 years, or had severe lung and heart diseases that affected lung function and who could not endure concurrent chemotherapy were excluded from this study. This study was authorized by the ethics committee [KS(Y)1636] according to the World Medical Association Declaration of Helsinki. Trial registration: Retrospective registration, ChiCTR1900027290(08/11/2019). The enrolled patients provided informed consent and agreed with the study.

### Patient assessment

To be eligible, all patients had to provide their medical history and undergo medical assessments such as a physical examination, routine blood test, renal and hepatic function tests, electrocardiography, chest contrast-enhanced computed tomography (CT), abdominal ultrasound or CT, whole-body bone scan, or 18F-fluorodeoxyglucose positron emission tomography (PET) CT, and brain magnetic resonance imaging (MRI) within 2 weeks before CRT. Eligible patients underwent at least one physical and routine blood examination weekly during the whole treatment.

### Radiotherapy

**Fig. 1:**
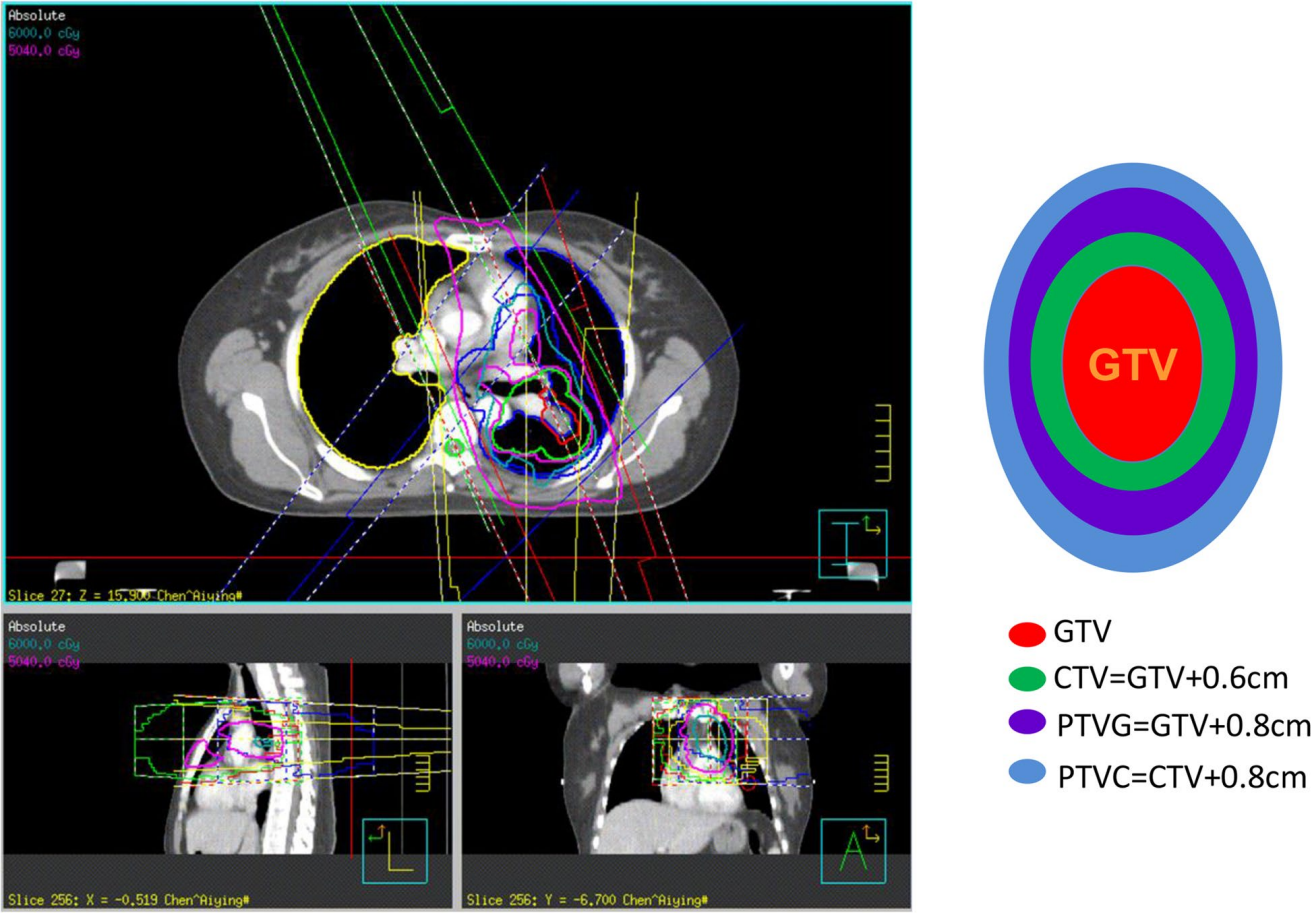
The margin and the prescribed dose of planning target volume (PTV). GTV: the gross tumor volume; CTV: The clinical target volume; PTVG: the expansion of GTV with a 0.8-cm margin; PTVC: the expansion of CTV with a 0.8-cm margin

The included patients underwent treatment simulation CT using an intravenous contrast in the supine position. The gross tumor volume (GTV) included the primary tumor and metastatic lymph nodes. GTV was contoured on a CT simulation scan by a radiation oncologist using various image sources of the pretreatment diagnostic CT scan and/or PET scan, pathological results of mediastinoscopy, and transthoracic CT or endobronchial ultrasound image-guided biopsies. Mediastinal lymph nodes measuring > 1 cm on diagnostic CT, positive result on pretreatment PET-CT, or positive biopsy results were all contained in the GTV. The clinical target volume (CTV) was delineated as the GTV with a 0.6-cm margin. The planning target volume (PTV) was the expansion of GTV with a 0.8-cm margin called PTVG, while PTV was the expansion of CTV with a 0.8-cm margin called PTVC (Fig. 1). The specific margin of patients was used to explain setup variation and internal organ motion.

Patients were enrolled for four levels of escalating radiation doses with SIB to GTV (Table 1). We chose conventionally fractionated radiation dose PTVG 2 Gy/Fx, 60 Gy/30 Fx, PTVC 1.8 Gy/Fx, and 54 Gy/30Fx as starting radiation doses (RTOG 7301 trial). Every dose level maintained the total physical dose essentially unchanged, increasing the dose per fraction. The requirements of minimal dose coverage were that 95% of PTVs received the prescription dose and 99% of PTVs received 95% of the prescription dose. The full course of hypofractionated radiation was conducted once per day at five fractions per week. Considering the lack of organs at risk (OAR) dose constraints for this fractionation regimen, we established dose-volume constraints for OAR as follows: (1) the maximum dose of the spinal cord was ≤45 Gy; (2) the percent volume of the total lung receiving > 20 Gy (V20) was ≤25%, (3) the mean lung dose (MLD) was ≤15 Gy, (4) the mean esophageal dose was ≤34 Gy, and (5) the mean heart dose (MHD) was ≤30 Gy.

**Table 1:** Dose Escalation

Dose LEVEL	PTV-G	PTV-C
**1**	60Gy/30Fx (2Gy/Fx)	54Gy/30Fx (1.8Gy/Fx)
**2**	60Gy/24Fx (2.5Gy/Fx)	50.4Gy/24Fx (2.1Gy/Fx)
**3**	60.5Gy/22Fx (2.75Gy/Fx)	49.5Gy/22Fx (2.25Gy/Fx)
**4**	60Gy/20Fx (3Gy/Fx)	50Gy/20Fx (2.5Gy/Fx)

### Chemotherapy

Chemotherapy and radiotherapy were initiated on the same day. Patients concurrently received six cycles of chemotherapy (cisplatin plus docetaxel). The doses of cisplatin and docetaxel were all 20 mg/m^2^ separately, one time every week, totally 6 weeks. Cisplatin was administered within 6 h. There was no consolidation chemotherapy after 6 weeks of chemotherapy cycles.

### Toxicity evaluation and dose escalation

Treatment toxicities were graded according to the National Cancer Institute Common Terminology Criteria for Adverse Events, version 4.0. Weekly toxicity was scored during concurrent CRT. Acute toxicity was defined as occurring within 3 months of initiating CRT, and late toxicities was defined as occurring later than 3 months. Dose-limiting toxicity (DLT) was defined as any grade 3 or higher acute and/or life-threatening toxicities that interrupted radiotherapy for more than 1 week.

Dose escalation was implemented according to the following rules. Each escalating dose level included a minimum of five patients. Toxicity was evaluated for all five patients at each dose level within a minimum of 3 months since initiating CRT. If DLT did not occur, the next dose level was administered. If one of the first five enrolled patients experienced DLT within 3 months, five additional patients were included at the same dose level. If a second patient experienced DLT or grade 5 toxicity at this level, dose escalation was discontinued.

### Dose modification

Modification of either radiotherapy or chemotherapy dosage was not permitted in this study. If patients experienced grade 3 or higher acute toxicities related to radiation, radiotherapy was delayed until the patient recovered. In contrast, if patients experienced acute toxicities unrelated to radiation, radiotherapy was implemented as planned; however, chemotherapy was discontinued until the toxicity was resolved within 2 weeks. If patients could not endure grade 3 or higher hematologic and non-hematologic toxicities, CRT was interrupted. If the treatment was interrupted for more than 2 weeks, the patient was withdrawn from the study.

### Endpoints

This study was a non-randomized, phase 1 clinic trial. The primary endpoint was MTD of hypofractionated SIB-IMRT with concurrent cisplatin/docetaxel chemotherapy. MTD was defined as the dose below which DLT or grade 5 toxicity occurred in two patients. The secondary endpoints were locoregional control, progression-free survival (PFS), and OS.

### Follow-up and statistics analysis

Every follow-up evaluation included a complete medical history, PS assessment, physical examination, routine blood test, chest CT, and abdominal ultrasound or CT. The first follow-up was 1 month after treatment, followed by every 3 months for 2 years and then every 6 months. Any significant treatment-related toxicity was also recorded. Further investigations such as brain MRI, whole-body bone scan, endoscopy, and biopsy were arranged as clinically suspected relapse of these sites.

Patients were followed for all protocol endpoints (including MTD and toxicity) indefinitely. Categorical variables are expressed as frequencies and percentages, and continuous variables are expressed as means, medians, standard deviations, and ranges. The survival time was calculated from the beginning of CRT to death or final follow-up using the Kaplan–Meier method. Statistical analyses were performed using the Statistical Package for Social Science 20.0 software (SPSS Inc., Chicago, IL, USA).

## Results

### Patients’ characteristics

From April 2012 to October 2013, 25 patients with previously untreated NSCLC who completed the prescribed radiation dose were included in this study. The patients’ characteristics are summarized in Table 2. Of 25 patients, 10 had squamous cell carcinoma, of whom 17 had stage IIIA carcinoma. Five patients each were enrolled for dose LEVEL 1–3 and 10 for dose LEVEL 4. The median dose volume of PTVs and parameters of OARs are provided in Table 3.

**Table 2:** Patient characteristics

Characteristic	Number	Percentage
**Gender**		
Female	6	24
Male	19	76
**Age**		
Median	60	
Range	38–72	
**Smoke Status**		
Moderate/heavy smoker	18	72
Nonsmoker/former light smoker	7	28
**Performance status**		
0	16	64
1	9	36
**Histology**		
Squamous cell carcinoma	10	40
Non-Squamous cell carcinoma	15	60
**Tumor location**		
Central	2	8
Left	11	44
Right	12	48
**Stage**		
IIIA	17	68
IIIB	8	32
**T Stage**		
T2	9	36
T3	11	44
T4	5	20
**N Stage**		
N2	18	72
N3	7	28
**Dose Level**		
1	5	20
2	5	20
3	5	20
4	10	40

**Table 3:** Parameters for the volumes and OARs

Dose Level	1	2	3	4
**GTV(cm**^**2**^**)**	51.0(45.4–309.1)	88.4(82.1–123.7)	73.81(41.22–106.79)	104.5(32.2–258.6)
**PTV-G(cm**^**2**^**)**	260.5(202.76–672.0)	298.7(242.5–374.0)	259.9(172.3–264.28)	324.2(159.6–663.3)
**CTV(cm**^**2**^**)**	138.7(114.3–568.7)	200.2(172.7–270.3)	198.1(105.6–292.2)	231.8(99.3–498.7)
**PTV-C(cm**^**2**^**)**	443.6(354.9–1020.6)	473.9(410.5–608.0)	386.1(288.6–623.9)	552.5(249.7–921.8)
**Normal Lung**				
V20 (%)	20.9(18.9–23.3)	18.9(16.7–23.2)	14.2(12.4–25.0)	22.24(16.9–28.4)
MLD (Gy)	12.2(10.4–13.0)	9.8(9.4–11.7)	8.23(6.5–12.7)	12.6(8.3–17.8)
V5%	44.54(34.9–57.5)	35.5(32.2–48.2)	37.8(23.9–51.7)	39.8(23.4–54.3)
V5%(ipsilateral)	23.0(10.1–30.9)	25.1(23.16–35.6)	24.7(16.56–38.7)	24.3(16.3–31.8)
V5%(contralateral)	59.7(58.3–60.2)	58.3(45.0–68.7)	61.48(52.063.54)	57.3(34.5–66.2)
**Heart**				
MHD (Gy)	12.49 (12.1–29.2)	13.3 (2.9–19.3)	12.1(4.9–16.9)	11.4 (6.0–27.0)
**Spinal cord**				
Dmax (Gy)	45.0 (39.7–48.0)	46.4 (31.5–48.9)	45.6(30.0–50.6)	43.9(4.9–47.7)
**Esophagus**				
Mean Dose (Gy)	28.8(15.2–37.0)	21.9 (10.1–50.0)	22.5(7.6–27.6)	24.0(4.9–39.4)

Data were collected as the median and rang

Of all 25 patients, the median GTV was 88.1 (mean 103.6, range 32.2–309.1) cm^3^, median CTV was 198.1 (mean 236.5, range 99.3–568.7) cm^3^, median PTVG was 283.5 (mean 330.8, range 159.6–672.0) cm^3^, and median PTVC was 473.9 (mean 527.1, range 249.7–1020.6) cm^3^. The detailed volume of all four dose levels is described in Table 3. The dose coverage of PTVs at each dose level met the defined requirement well.

### Toxicity and MTD

All 25 patients underwent toxicity and efficacy evaluations; the number of patients at all 4 levels is shown in Table 4. During the period of concurrent treatment, approximately 56% of patients in this study had grade 1–2 hematologic toxicities. The most common hematologic toxicities were grade 1–2 neutropenia and anemia. Only one patient receiving dose LEVEL 4 had grade 3 hematologic toxicity (febrile neutropenia) and successfully recovered from the toxicities within 3 days with granulocyte-colony-stimulating factor (G-CSF) and supportive care. Grade 3 hematologic toxicities of this study were manageable and reversible. In total, hematological toxicities from weekly cisplatin plus docetaxel treatments were well tolerated. Only one patient of the first five patients receiving dose LEVEL 4 could not endure grade 3 gastrointestinal toxicity, and thus, the sixth weekly dose of concurrent chemotherapy was canceled. All other patients completed six cycles of weekly chemotherapy.

**Table 4:** Toxicity

Toxicity	Grade I- II	Grade III	GradeIV -V
**Dose LEVEL1 (5pts)**			
Hematologic toxicity	3		
Gastrointestinal toxicity	2		
Radiation Esophagitis	2	1	
Radiation Pneumonitis	4		
**Dose LEVEL2 (5pts)**			
Hematologic toxicity	2		
Gastrointestinal toxicity	2		
Radiation Esophagitis	2	1	
Radiation Pneumonitis	2		
**Dose LEVEL3 (5pts)**			
Hematologic toxicity	3		
Gastrointestinal toxicity	1		
Radiation Esophagitis	3		
Radiation Pneumonitis	3		
**Dose LEVEL4 (10pts)**			
Hematologic toxicity	6	1	
Gastrointestinal toxicity	4	2	
Radiation Esophagitis	5		1
Radiation Pneumonitis	5		1

The most common radiation toxicities were grade 1–2 radiation esophagitis (35%) and pneumonitis (55%) (Table 4). Only one patient each had grade 3 acute radiation esophagitis in dose LEVEL 1–2. However, this radiation-related toxicity was successfully alleviated by providing the best supportive care without interrupting the implementation of concurrent CRT. Only one patient receiving dose LEVEL 2 experienced an interruption of radiotherapy because of a 3-day fever, and all others completed CRT as planned without interruption. No patients developed DLT in dose LEVEL 1–3. In contrast, two patients experienced grade 5 toxicities in dose LEVEL 4. The first patient experienced grade 3 nausea and vomiting, failed to complete the six chemotherapy cycles, and died because of upper gastrointestinal hemorrhage within 6 months after the treatment. To ensure safety at the same level, five additional patients were enrolled. One of the second five patients also had grade 5 radiation pneumonitis and died within 2 months post-radiation after receiving the best treatment and supportive care. The lung dose-volume constraints of this patient were as follows: V20 of 28.0% and MLD of 15.8 Gy. Therefore, dose LEVEL 3 (PTVG 60.5 Gy/22 Fx, 2.75 Gy/Fx; PTVC 49.5 Gy/22 Fx, 2.25 Gy/Fx) was regarded as the MTD of hypofractionated SIB-IMRT concurrent with chemotherapy. The study was subsequently closed.

### Treatment efficacy and survival

Among the included 25 patients, the response rate, compete response rate, partial response rate, and stable disease rate were 92.0% (23/25), 4.0% (1/25), 88.0% (22/25), and 8.0% (2/25), respectively. None of the patients had progressive disease.

**Fig. 2:**
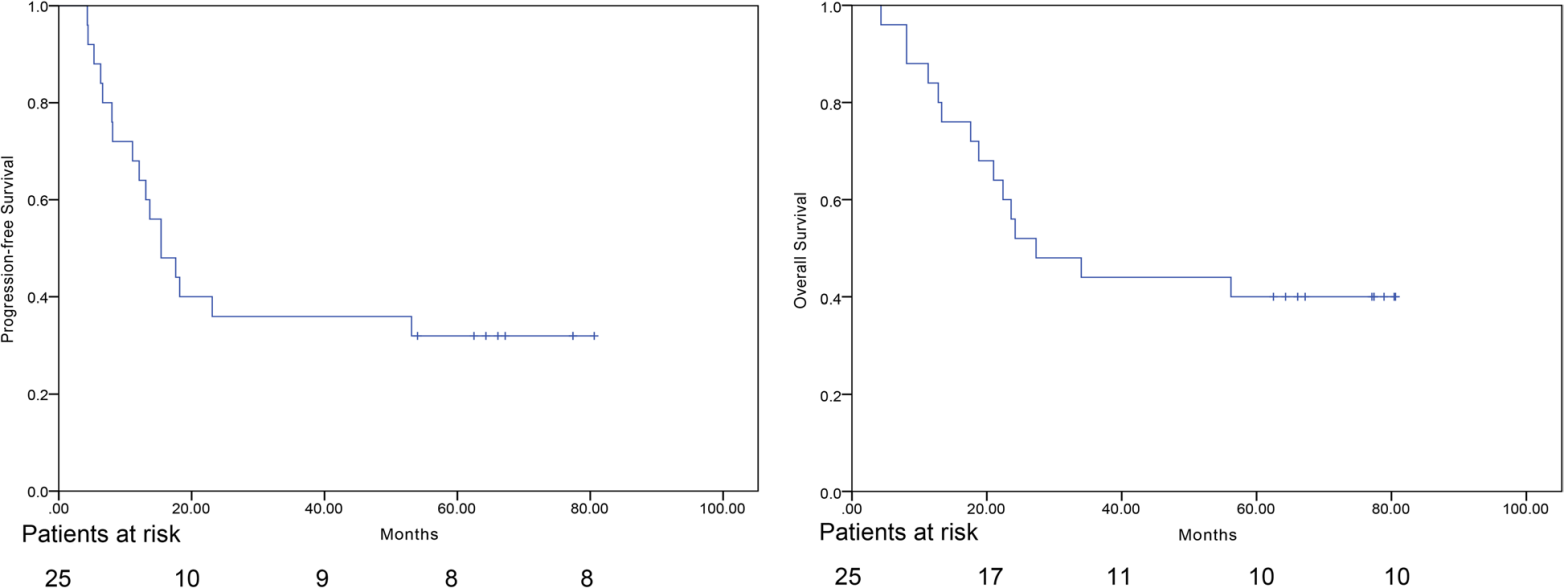
The median follow-up time was 77.1 (range 4.3–80.6) months. The median progression-free survival (PFS) and overall survival (OS) were 15.4 and 27.3 months

The median follow-up time was 77.1 months (4.3–80.6 months). At the time of analysis, 10 patients were still alive with disease and eight were progression free. Of 15 patients who experienced recurrence as the first failure, three had local failure in the radiation field, 10 had distant failure, and two had both recurrences outside of the radiation field and distant metastases simultaneously. The 1- and 2-year locoregional control rates were 82.8 and 67.8%. The median PFS was 15.4 months, and the 1- and 3-year PFS rates were 64.0 and 34%, respectively. The median OS was 27.3 months, and the 1- and 3-year OS rates were 84.0 and 44.0%, respectively. PFS and OS over time are shown in Fig. 2.

## Discussion

The optimal radiation dose and fraction for unresectable stage III NSCLC have been a major research challenge for a long time. Radiation Therapy Oncology Group (RTOG) 0617 could not demonstrate the survival benefit using a high dose of 74 Gy. A longer treatment time, higher cardiac dose, and lower proportion of concurrent chemotherapy were considered responsible for the worse OS in the 74-Gy group of RTOG 0617. The potential survival benefit of hypofractionated radiation for unresectable NSCLC has been reported in several studies: Kim et al. conducted a prospective dose-escalation study of hypofractionated radiation with concurrent chemotherapy for unresectable or inoperable NSCLC (up to 48 Gy/20 Fx plus 22.7 Gy/7 Fx, EQD2 ≈ 92 Gy/46 Fx), and the results proved that the hypofractionated treatment was well tolerated and had better locoregional control [28]; Amini et al. reported similar results, certifying that hypofractionated radiation can improve treatment efficacy with a shorter treatment time [29]; An Italian prospective investigation also reported additional evidence that hypofractionated radiation (60 Gy/20 Fx) was well tolerated and safe for inoperable advanced-stage NSCLC [30]. Compared with conventional fractionation, hypofractionation is an economical and convenient treatment with easy implementation and is applied more often in clinical settings. This prospective radiation dose escalation study determined the MTD of hypofractionated radiation concurrent with chemotherapy using SIB-IMRT for unresectable stage III NSCLC patients. The dose of GTV could increase by 22% using SIB-IMRT without significant changes in doses on the surrounding normal tissue [31]. SIB-IMRT may be better in dose escalation of a target tumor, whereas OAR remain in safe dose-volume constraints.

As this study showed, the dose escalation schedule was conducted based on both radiobiological and practical rationales. According to the conventional 2-Gy per fraction regimen, the total physical dose remained essentially unchanged, and using hypofractionated escalation of the radiation dose, the whole treatment period was shortened and the BED was significantly increased. Dose escalation was safely achieved to dose LEVEL 3 (PTVG 60.5 Gy/22 Fx, 2.75 Gy/Fx; PTVC 49.5 Gy/22 Fx, 2.25 Gy/Fx) with SIB-IMRT. DLT did not occur in the light of our definition until dose LEVEL 4. Two patients died at this level. One patient died because of upper gastrointestinal hemorrhage; however, whether the death was related to radiation toxicity or the patient’s own underlying upper gastrointestinal disease was unclear. However, another death in the additional five patients was observed at the same level. This patient with a T4N2M0 tumor and a relatively large GTV had grade 5 radiation pneumonitis. The same result was also reported in the Korean Radiation Oncology Group 0301 study using a 3D conformal GTV simultaneous boost (60 Gy, 2.4 Gy/Fx) in CRT for patients with LA-NSCLC [32]. These severe toxicities highlight the importance of carefully screening patients for hypofractionated radiation in future dose escalation phase I or II trials. All grade 1–3 radiation-related toxicities were recoverable with supportive care, and the patients receiving dose LEVEL 1–3 completed the planned CRT without interruption. Only one patient receiving dose LEVEL 4 experienced interruption of radiotherapy because of fever that lasted for 3 days, which seemed unrelated to dose escalation. During the follow-up, all acute toxicities were acceptable, and no further late toxicity was observed. However, late toxicities still needed further observation because the risk of damages increased as the dose escalated; therefore, dose LEVEL 3 (PTVG 60.5 Gy/22 Fx, 2.75 Gy/Fx; PTVC 49.5 Gy/22 Fx, 2.25 Gy/Fx) was the MTD. We are currently conducting a phase II clinical trial to evaluate long-term outcomes and late toxicities of LEVEL 3 dose in a larger sample size.

Platinum-based concomitant CRT is the standard care for LA-NSCLC; however, the optimal chemotherapy drugs are unclear [4, 33]. In this study, the concurrent regimens were weekly cisplatin and docetaxel. Except for one patient, everyone else (24/25, 96%) completed six weekly scheduled cycles of chemotherapy. The poor outcome of this one patient was primarily owing to intolerable grade 3 nausea in dose LEVEL 4. In the RTOG 0617 randomized controlled trial, the completion of concurrent chemotherapy delivery was 88% in the conventional 60-Gy dose group and 85% in the higher 74-Gy dose group [6]. Weekly cisplatin and docetaxel administration owing to their completion and favorable toxicity can be recommended as chemotherapy drugs with concurrent CRT for unresectable stage III NSCLC.

In the present study, the 1-year locoregional control was 82.8%, and the median PFS and OS were 15.4 and 27.3 months, respectively, which are similar to previous study results [6, 7]. The two patients who had DLT and died after treatment in this study could have affected the survival of the entire study group. In the PACIFIC study of unresectable stage III NSCLC, the PFS was 11 months longer for patients who received durvalumab after CRT than in placebo patients, the PFS and OS were 16.9 VS 5.6. months and 47.5 VS 29.1 months, sepetately [34–36]. Several radiation studies have reported that radiation has an advantage for immune activation [36–39]. The selection of optimal radiation dose and fraction has a key role in immune activation. Subgroup analysis of the PACIFIC study showed that the group of dose <60Gy also had the similar PFS and OS as the high dose group. The antitumor immune responses were inclined to be triggered by hypofractionated radiation in some preclinical experiments [36–39]. More and more evidences supported that hypofractionated radiation can get better survival combined with immunotherapy, such as Pembro-RT and Bauml study. But we need more prospective clinical data about unresectable stage III NSCLC. Regardless, our study results supported the feasibility of hypofractionated radiation using SIB-IMRT, and thus, further research should focus on whether this hypofractionated radiation, not only combined with chemotherapy but also with immunotherapy, is better than the conventional fraction regimen based on OS and toxicities.

This study had some limitations. First, DLT was measured based on acute toxicities and not late toxicities or OS. Second, the MHD was ≤30 Gy, which was significantly higher than the 20 Gy recommended by the National Comprehensive Cancer Network guidelines. However, in this study, the incidences of cardiotoxicities were not obviously increased during the observation period. Finally, the standard therapy for unresectable stage III NSCLC is concurrent CRT combined with durvalumab; however, it is unclear whether the dose escalation using SIB-IMRT in the era of chemotherapy drugs combined with immunotherapy is still safe and effective.

## Conclusion

In the era of chemotherapy drugs, our dose escalation protocol was successful at dose LEVEL 3 (PTVG 60.5 Gy/22 Fx, 2.75 Gy/Fx; PTV-C 49.5 Gy/22 Fx, 2.25 Gy/Fx) using SIB-IMRT for unresectable stage III NSCLC, and the acute toxicities were all acceptable. It appears to be an effective and safe therapeutic approach for unresectable stage III NSCLC. Hence, further studies should assess long-term clinic outcomes and late toxicities and also examine whether dose escalation using SIB-IMRT combined with immunotherapy is suitable.

## Supplementary Information

**Additional file 1.**

**Additional file 2.**

## Data Availability

The datasets generated and/or analysed during the current study are not publicly available due to part of the data in this study related to other related studies, but are available from the corresponding author on reasonable request.
